# Tiotropium modulates transient receptor potential V1 (TRPV1) in airway sensory nerves: A beneficial off-target effect?^⋆^^☆^

**DOI:** 10.1016/j.jaci.2013.12.003

**Published:** 2014-03

**Authors:** Mark A. Birrell, Sara J. Bonvini, Eric Dubuis, Sarah A. Maher, Michael A. Wortley, Megan S. Grace, Kristof Raemdonck, John J. Adcock, Maria G. Belvisi

**Affiliations:** Respiratory Pharmacology, National Heart and Lung Institute, Faculty of Medicine, Imperial College London, London, United Kingdom

**Keywords:** Sensory nerves, vagus, cough, ion channels, capsaicin, anticholinergics, [Ca^2+^]_i_, Intracellular calcium, COPD, Chronic obstructive pulmonary disease, DiI, DilC18(3)-1,1′-dioctacetyl-3,3,3′,3′-tetramethyl-indocarbocyanine perchlorate, DMSO, Dimethyl sulfoxide, ECS, Extracellular solution, K_50_, 50 mmol/L potassium chloride extracellular solution, LAMA, Long-acting muscarinic receptor antagonist, MCh, Methacholine, Penh, Enhanced pause, PGE_2_, Prostaglandin E_2_, RTX, Resiniferatoxin, TRP, Transient receptor potential, URI, Upper respiratory tract infection

## Abstract

**Background:**

Recent studies have suggested that the long-acting muscarinic receptor antagonist tiotropium, a drug widely prescribed for its bronchodilator activity in patients with chronic obstructive pulmonary disease and asthma, improves symptoms and attenuates cough in preclinical and clinical tussive agent challenge studies. The mechanism by which tiotropium modifies tussive responses is not clear, but an inhibition of vagal tone and a consequent reduction in mucus production from submucosal glands and bronchodilation have been proposed.

**Objective:**

The aim of this study was to investigate whether tiotropium can directly modulate airway sensory nerve activity and thereby the cough reflex.

**Methods:**

We used a conscious cough model in guinea pigs, isolated vagal sensory nerve and isolated airway neuron tissue– and cell-based assays, and *in vivo* single-fiber recording electrophysiologic techniques.

**Results:**

Inhaled tiotropium blocked cough and single C-fiber firing in the guinea pig to the transient receptor potential (TRP) V1 agonist capsaicin, a clinically relevant tussive stimulant. Tiotropium and ipratropium, a structurally similar muscarinic antagonist, inhibited capsaicin responses in isolated guinea pig vagal tissue, but glycopyrrolate and atropine did not. Tiotropium failed to modulate other TRP channel–mediated responses. Complementary data were generated in airway-specific primary ganglion neurons, demonstrating that tiotropium inhibited capsaicin-induced, but not TRPA1-induced, calcium movement and voltage changes.

**Conclusion:**

For the first time, we have shown that tiotropium inhibits neuronal TRPV1-mediated effects through a mechanism unrelated to its anticholinergic activity. We speculate that some of the clinical benefit associated with taking tiotropium (eg, in symptom control) could be explained through this proposed mechanism of action.

Inhaled muscarinic receptor antagonists are currently used as bronchodilators for the management of asthma and chronic obstructive pulmonary disease (COPD).^1^ Their efficacy is believed to be based on the notion that they block increased vagal tone (through increased parasympathetic cholinergic contractile responses, acetylcholine release, and muscarinic receptor activation on airway smooth muscle), which is thought to be the major reversible component of airflow narrowing in patients with COPD.^2,3^ Tiotropium was the first long-acting muscarinic receptor antagonist (LAMA), reaching the market in 2002.^4-6^ Initially, tiotropium was prescribed for its bronchodilator effects in human subjects, but more recent evidence suggests that it might also be effective in improving patients' quality of life, reducing exacerbations, and increasing exercise capacity.^7^ Tiotropium has also recently been shown to improve asthma symptoms and lung function in patients with inadequately controlled asthma.^8^

Preclinical and clinical studies have also suggested that tiotropium inhibits cough in tussive challenge models.^9,10^ Dicipinigiatis et al^10^ reported that tiotropium inhibits capsaicin (transient receptor potential [TRP] V1 agonist)–induced cough in patients with upper respiratory tract infections (URIs) in a prospective, randomized, double-blind, placebo-controlled clinical trial. Bouyssou et al^9^ reported that tiotropium caused a dose-dependent inhibition of citric acid–induced cough in a guinea pig asthma model. An antitussive action of tiotropium in acute challenge models through its antimuscarinic activity is difficult to conceive. Various explanations have been proffered, including inhibition of vagal tone (and thereby mucus secretion and bronchodilation) elicited through blockade of muscarinic M_3_ receptors on submucosal glands and airway smooth muscle, respectively. However, the mechanism behind the antitussive activity has never been fully elucidated.

The aim of this study was to investigate whether tiotropium can directly modulate airway sensory nerves and thereby tussive responses by using a range of techniques. We used an isolated vagus nerve preparation and calcium imaging of primary sensory jugular neurons to circumvent the potentially confounding bronchodilator, anti-inflammatory, and antimucolytic properties of tiotropium, which are presumably associated with its antimuscarinic activity, and to avoid the pharmacokinetic and numerous other considerations that limit the interpretation of *in vivo* data. The ability to use human vagus nerve preparations also allowed us the opportunity to translate our findings to the clinical setting. An inhibitory activity on capsaicin-induced action potential firing *in vivo* confirmed an interaction of tiotropium with TRPV1 on airway-specific C-fibers.

In summary, our data suggest that tiotropium inhibits TRPV1 ion channel activity through a mechanism unrelated to its anticholinergic activity. This activity is not through a general inhibition of sensory nerve activity because TRPA1-mediated responses were not affected. In conclusion, we suggest that some of the clinical benefit associated with taking tiotropium could be explained through its inhibition of TRPV1 responses.

## Methods

### Effect of tiotropium on capsaicin-induced cough

To establish an effective dosing regimen, we first performed a concentration response to inhaled tiotropium against methacholine (MCh)–induced bronchospasm (as estimated by changes in enhanced pause [Penh]). Conscious guinea pigs were exposed to either aerosolized vehicle (0.5% ethanol in saline) or tiotropium (3, 10, or 30 μg/mL; this equates to 6.35, 21.2, and 63.5 μmol/L solution) for 10 minutes and were challenged 50 minutes later with either saline or MCh (0.1 μg/mL). Changes in Penh were recorded for 5 minutes. From these data, doses of tiotropium were selected to be tested against capsaicin-induced cough, as previously described.^11-14^ Briefly, after exposure to vehicle or tiotropium solution as above, cough was induced by exposing the guinea pigs to an aerosol of capsaicin (60 μmol/L) for 5 minutes. See additional methods in the Methods section in this article's Online Repository at www.jacionline.org.

### Effect of tiotropium on isolated vagal sensory nerve tissue

Guinea pigs were culled with an overdose of pentobarbitone (200 mg/kg administered intraperitoneally). The 2 vagal trunks were carefully dissected free and placed in Krebs-Henseleit solution. The segments of vagus nerve were mounted in a grease-gap dual-recording chamber system, as previously described, and depolarization (as an indicator of sensory nerve activity) of the nerve was assessed.^11-14^ Briefly, tissue was exposed to pre-established submaximal concentrations of the TRP agonist twice, treated with vehicle or test compound, and then rechallenged with the TRP agonist. After a wash step, the TRP agonist was reapplied. The effect of tiotropium was investigated on depolarization induced by a range of TRPV1 agonists, including capsaicin,^13,15^ and against depolarization induced by the TRPA1 agonist acrolein (300 μmol/L)^12^ and the TRPV4 agonist GSK1016790A (0.3 μmol/L).^16^ Key experiments were repeated with human vagal tissue. Ethical approval to use recipient human lung/vagal tissue (transplant tissue) was obtained from the Royal Brompton & Harefield Trust (REC reference 09/H0708/72). See additional methods in the Methods section in this article's Online Repository.

### Effect of tiotropium on airway-specific ganglion cells

Identification of airway-specific neurons was performed, as previously described.^17,18^ Briefly, 14 days before the experiment, guinea pigs were dosed intranasally with the lipophilic retrograde tracer dye DilC18(3)-1,1′-dioctacetyl-3,3,3′,3′-tetramethyl-indocarbocyanine perchlorate (DiI). Guinea pigs were then killed, and the jugular ganglia were harvested to measure calcium movement and membrane voltage change, as described previously.^13^ DiI-labeled neurons from jugular ganglia were then stained with both a ratiometric calcium-sensitive dye (Fura2-AM, 3 μmol/L) and a voltage-sensitive dye (Di-8-ANEPPS). The focus was on jugular ganglion cells because we have previously found these to be more responsive to capsaicin under normal conditions compared with airway nodose ganglion cells.^13^ The responsiveness and viability of neurons were assessed by means of application of 50 mmol/L potassium chloride extracellular solution (K_50_) at the start and end of recording. Intracellular calcium ([Ca^2+^]_i_) responses were recorded as the area under the curve, and membrane depolarization responses were recorded as the peak magnitude. All responses were normalized to the initial K_50_ application. See additional methods in the Methods section in this article's Online Repository.

### Effect of tiotropium on capsaicin-driven TRPV1 FLIPR assays (performed with GenScript)

HEK293 cells were genetically modified to overexpress human TRPV1 and seeded in a 384-well, black-wall, clear-bottom plate at a density of 20,000 cells per well in 20 μL of medium. Cells were cultured for 18 hours before the day of the experiment and maintained at 37°C in 5% CO_2_. Capsaicin concentration-response curves were performed to select a submaximal concentration in HEK293 cells overexpressing human TRPV1. Calcium-4, in conjunction with a Fluorescent Imaging Plate Reader, was used to record the signal. Assays were performed in duplicate. See additional methods in the Methods section in this article's Online Repository.

### Effect of tiotropium on capsaicin-induced firing of single-fiber afferents and bronchospasm

Guinea pigs were anesthetized with urethane (1.5 g/kg) intraperitoneally. The trachea was cannulated, and bronchospasm was measured with an air-pressure transducer connected to a side arm of the tracheal cannula. Animals were paralyzed with vecuronium bromide, which was initially administered at an intravenous dose of 0.10 mg/kg and followed every 20 minutes with 0.05 mg/kg administered intravenously to maintain paralysis. Firing of single-fiber afferents and bronchospasm was measured, as previously described.^19^ Briefly, after the vagus nerve was dissected clear of tissue, a single fiber was isolated and placed on the recording electrodes. After establishing it was an airway C-fiber, the animal was challenged with inhaled capsaicin after vehicle or inhaled test compound, and action potentials were recorded. See additional methods in the Methods section in this article's Online Repository.

### Effect of tiotropium on isolated guinea pig tracheal contractions

Contractile responses to MCh or capsaicin were induced in isolated guinea pig trachea by using a system previously described.^20^ Briefly, the trachea was placed in oxygenated Krebs-Henseleit solution at 37°C and exposed to cumulative doses of stimulant in the presence of vehicle or test compound, and contraction was assessed. See additional methods in the Methods section in this article's Online Repository.

### Data analysis and statistics

Inhibition of capsaicin-induced cough was analyzed by using the nonparametric Kruskal-Wallis test with the Dunn *post hoc* test, comparing drug group responses with those of the vehicle control group. Data are presented as medians ± interquartile ranges. In the *in vivo* single-fiber experiments, inhibition was determined by using a 2-tailed paired Student *t* test, comparing responses to agonist (in the same fiber) in the presence and absence of vehicle/antagonist. Inhibition of agonist-induced vagus nerve depolarization was analyzed by using the 2-tailed paired Student *t* test, comparing responses to agonist (in the same piece of vagus nerve) in the absence and presence of vehicle/antagonist. For imaging, all responses in each cell were normalized to the initial response generated by application of K_50_ within the same cell. Data were analyzed for “responding” cells only and defined as a neuron with a response of 10% or greater of K_50_. Inhibition of responses was analyzed by using the 2-tailed Student paired *t* test, comparing responses to agonist (in the same cell) in the absence and presence of vehicle/antagonist. Data are presented as means ± SEMs. For all statistical analyses, a *P* value of less than .05 was considered significant.

## Results

### Effect of tiotropium on capsaicin-induced cough

**Fig E1:**
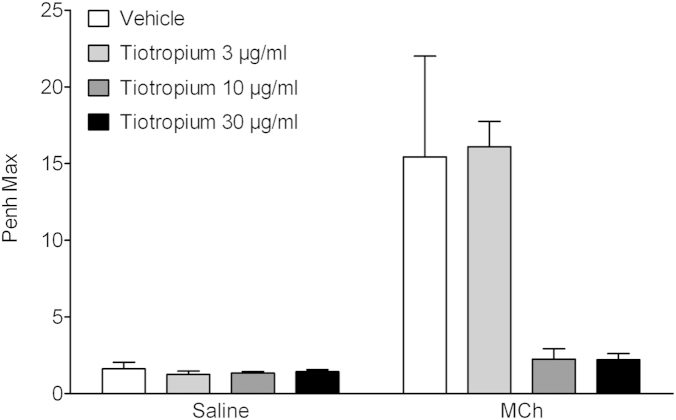
Effect of inhaled tiotropium on MCh-induced Penh change. Conscious guinea pigs were exposed to an aerosol of vehicle (0.5% ethanol in saline) or tiotropium (3, 10, or 30 μg/mL) for 10 minutes. Fifty minutes later, the guinea pigs were placed in the chambers used for cough experiments and challenged with an aerosol of saline or MCh (0.1 μmol/L). Penh (a noninvasive surrogate measurement of bronchospasm) was assessed by using Buxco software. Data are shown as means and SEMs (n = 3).

**Fig 1:**
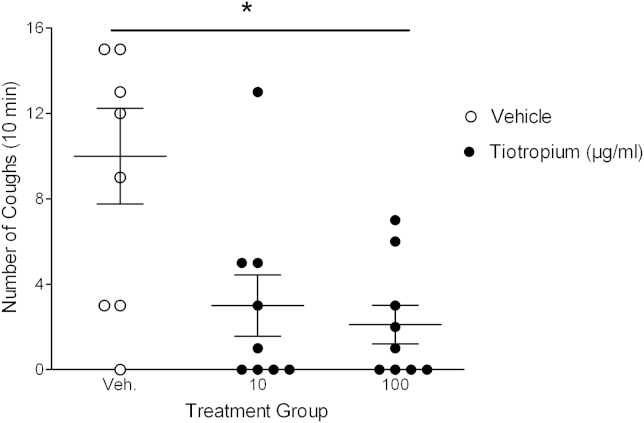
Effect of inhaled tiotropium on capsaicin-induced cough. Conscious guinea pigs were exposed to an aerosol of vehicle (0.5% ethanol in saline) or tiotropium (10 or 100 μg/mL) for 10 minutes. Fifty minutes later, the guinea pigs were challenged with an aerosol of capsaicin (60 μmol/L) for 5 minutes. Cough was recorded during the 5-minute challenge and for a further 5 minutes. Data are shown as means and SEMs (n = 9). *P < .05 as determined by Kruskall-Wallis with Dunn *post hoc* analysis.

Aerosolized tiotropium caused a dose-related blockade of MCh-induced change in Penh (a noninvasive surrogate measurement of bronchoconstriction) in the conscious guinea pig (see Fig E1 in this article's Online Repository at www.jacionline.org). From these data, we selected the lowest effective dose of tiotropium (10 μg/mL ≈ 21.2 μmol/L) and a log-fold higher dose (because we did not know the potency and pharmacokinetic profile of tiotropium against the sensory nerve target) to test against capsaicin-induced cough. Tiotropium caused a significant inhibition of capsaicin-induced cough (Fig 1).

### Effect of tiotropium on isolated vagal sensory nerve tissue

**Fig E2:**
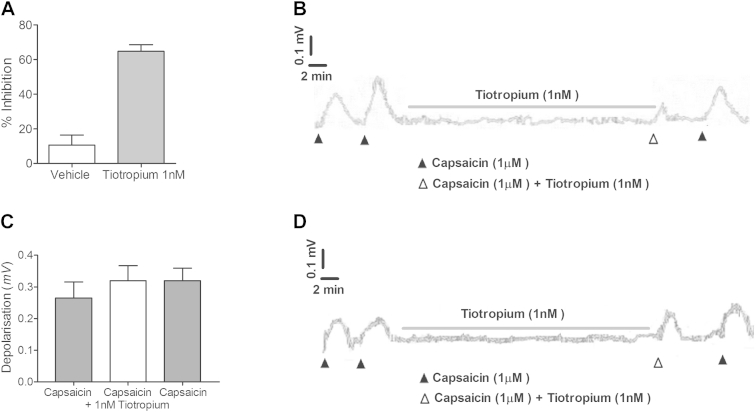
Effect of tiotropium on inhibition of capsaicin responses on isolated rat and mouse vagal tissue. The vagal tissue was challenged twice with capsaicin (1 μmol/L) to evoke 2 consecutive control depolarization responses, and then the tissue was exposed to vehicle or tiotropium (1 nmol/L) for 30 minutes. The tissue was then exposed to capsaicin in the presence of tiotropium. After a washout, the tissue was rechallenged with capsaicin to confirm tissue viability. **A,** Inhibition of depolarization induced by capsaicin (1 μmol/L) in rat tissue. **B,** An example trace. **C,** Depolarization (mV) of mouse isolated vagus nerve tissue induced by capsaicin or capsaicin in the presence of tiotropium and recovery capsaicin response, indicating that there is no inhibition of the response in mouse tissue. **D,** An example trace in mouse tissue.

**Fig E3:**
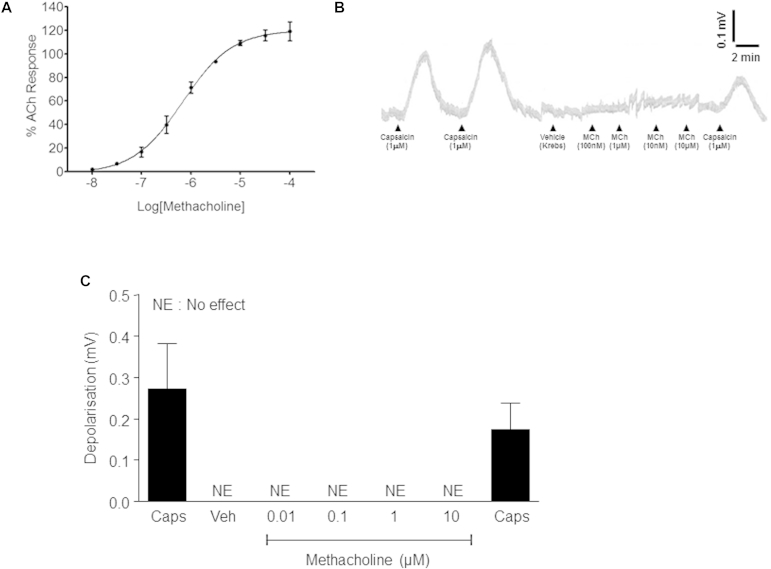
Effect of MCh on isolated guinea pig trachea and vagal tissue. The MCh cumulative response curve was performed. **A,** MCh caused a concentration-related increase in tension (expressed as a percentage of the average acetylcholine *[ACh]* response; n = 4). The vagal tissues were exposed to capsaicin (1 μmol/L) twice before they were challenged with vehicle or various concentrations of MCh. **B,** An example vagal trace. **C,** Average response over the 4 vagus nerve samples.

**Fig 2:**
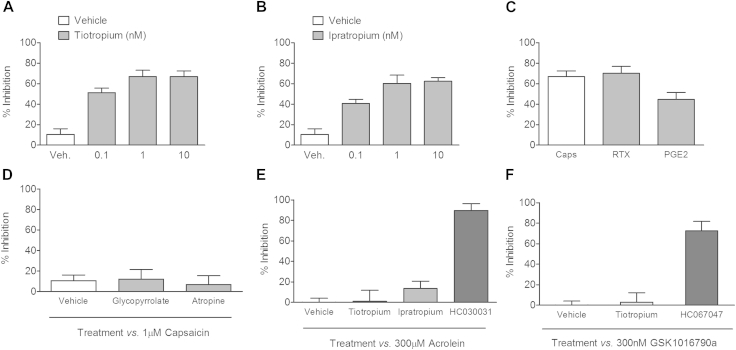
Effect of muscarinic antagonists on depolarization of isolated guinea pig vagus nerve. **A** and **B,** Concentration response data testing tiotropium and ipratropium against capsaicin (1 μmol/L)–induced depolarization. **C,** Effect of tiotropium (1 nmol/L) against a range of TRPV1 agonists (capsaicin, 1 μmol/L; RTX, 3 nmol/L PGE_2_, 10 μmol/L). **D,** Glycopyrrolate (10 nmol/L) and atropine (1 μmol/L) were tested against capsaicin responses. **E** and **F,** Tiotropium (1 nmol/L) and ipratropium (10 nmol/L) were profiled against acrolein (TRPA1 agonist, 300 μmol/L)–induced depolarization, with HC-030031 (TRPA1 antagonist, 10 μmol/L) as a positive control (Fig 2, *E*), and tiotropium was also profiled against GSK1016790a (TRPV4 agonist, 300 nmol/L)–induced depolarization, with HC-067047 (TRPV4 antagonist, 10 μmol/L) as a positive control (Fig 2, *F*). Data are shown as means ± SEMs of the percentage inhibition of tussive agent–induced depolarization (n = 4-6).

**Fig 3:**
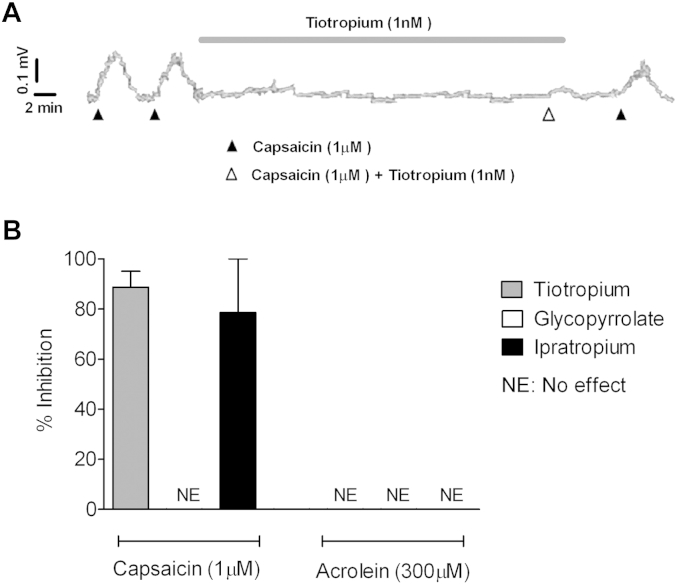
Effect of muscarinic antagonists on depolarization of isolated human vagus. **A,** Example trace from human vagal tissue. **B,** Inhibitory effect of tiotropium (1 nmol/L), ipratropium (10 nmol/L), and glycopyrrolate (10 nmol/L) when tested against capsaicin (1 μmol/L)– and acrolein (300 μmol/L)–induced depolarization. Data are shown as means ± SEMs of the percentage inhibition of tussive agent–induced depolarization (n = 2-3).

Tiotropium and a structurally similar muscarinic antagonist, ipratropium, caused a concentration-dependent inhibition of capsaicin-induced depolarization in the isolated guinea pig vagus nerve (Fig 2, *A* and *B*). This inhibition was also seen in the rat isolated vagus nerve, where tiotropium (1 nmol/L) inhibited capsaicin-induced depolarization. However, tiotropium did not cause any inhibition of capsaicin responses in the mouse vagus nerve (see Fig E2 in this article's Online Repository at www.jacionline.org). To demonstrate that the effect of tiotropium was not specific to capsaicin-induced activation of TRPV1, we showed that it modulated a second TRPV1 selective agonist, resiniferatoxin (RTX), as well as prostaglandin E_2_ (PGE_2_), which is known to act partially through the TRPV1 channel (Fig 2, *C*).^13^ Interestingly, other muscarinic receptor antagonists (glycopyrrolate and atropine) did not inhibit capsaicin responses (Fig 2, *D*). Tiotropium and ipratropium did not modulate TRPA1 or TRPV4 agonist–induced depolarization of guinea pig vagus. In these experiments the specific TRPA1 and TRPV4 antagonists HC-030031 and HC-067047, respectively (10 μmol/L), were used as positive controls (Fig 2, *E* and *F*). MCh caused a concentration-related contraction of guinea pig tracheal tissue but did not induce depolarization of the vagal tissue (see Fig E3 in this article's Online Repository at www.jacionline.org). Both tiotropium and ipratropium attenuated capsaicin-induced, but not acrolein-induced, depolarization of human vagal tissue, whereas glycopyrrolate was ineffective (Fig 3).

### Effect of tiotropium on changes in [Ca^2+^]_i_ and membrane voltage in primary guinea pig sensory jugular neurons

**Fig E4:**
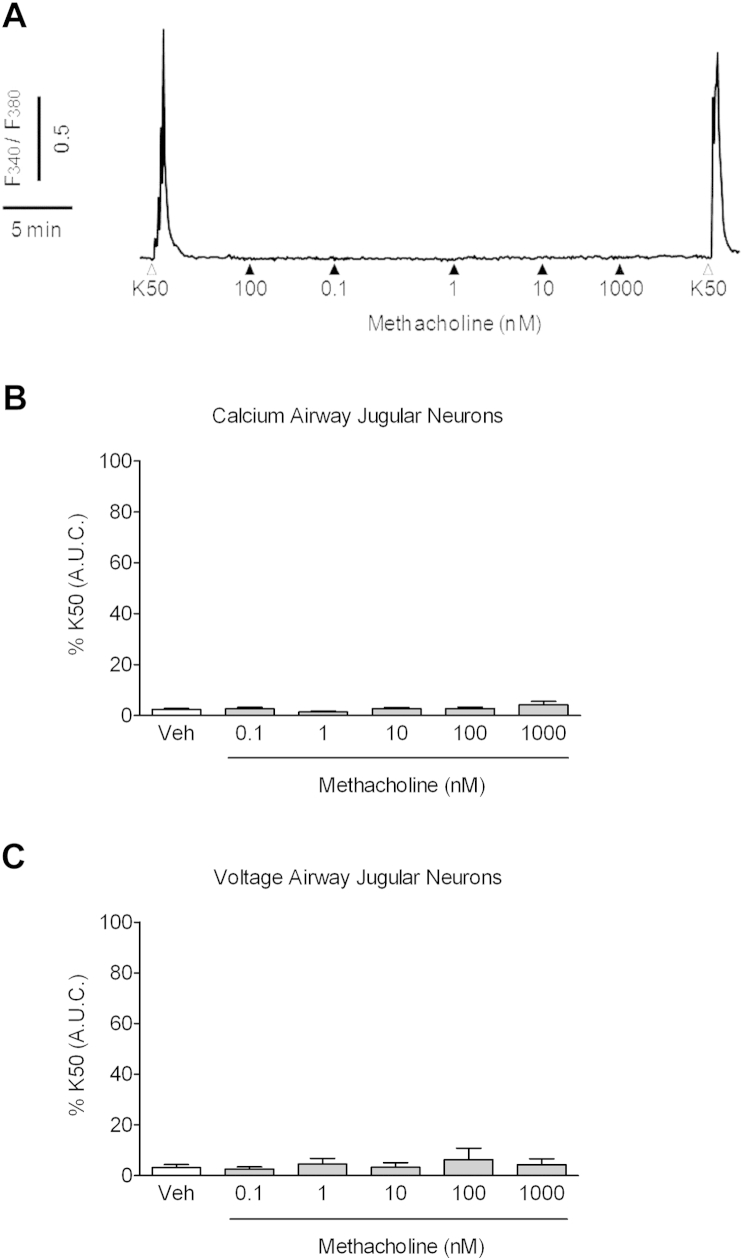
Effect of MCh on primary airway guinea pig ganglion cells. Guinea pig airway-specific jugular ganglion neurons were challenged with K_50_ for a reference response and then exposed to vehicle or random concentrations of MCh, and calcium levels and changes in membrane voltage were recorded. **A,** An example of a calcium recording. **B** and **C,** Mean calcium and voltage changes in cells (7 cells harvested from 3 guinea pigs). *A.U.C*., Area under the curve.

**Fig 4:**
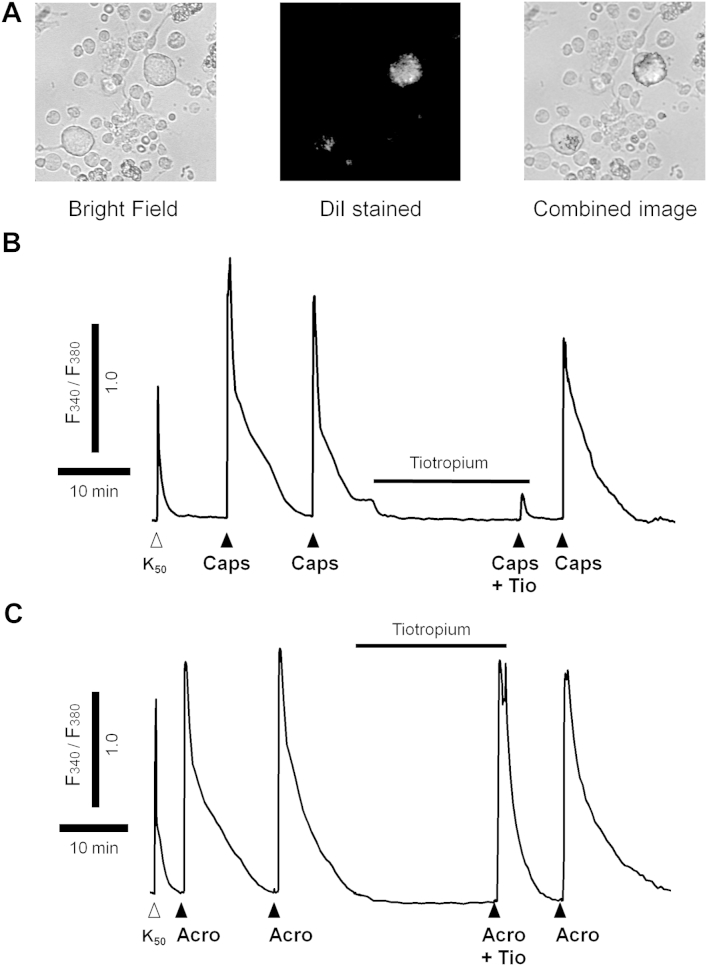
Effect of tiotropium on capsaicin- and acrolein-induced [Ca^2+^]_i_ in primary airway guinea pig ganglion cells. Airway-specific neurons were chosen by using the Dil stain as a marker; an example is shown in **(A)**. Neurons were challenged with K_50_ for a reference response and then exposed to 2 challenges of either capsaicin (*Caps*, 0.1 μmol/L) or acrolein (*Acro*, 10 μmol/L). The cells were then exposed to 0.1 nmol/L tiotropium for 20 minutes, after which the cells were rechallenged with the respective challenge agent. After a wash step, the cells were challenged with the tussive agent. **B** and **C,** Example traces were obtained: *open triangles* indicate K_50_ challenge, and *solid triangles* show where the challenge agent was added.

**Fig 5:**
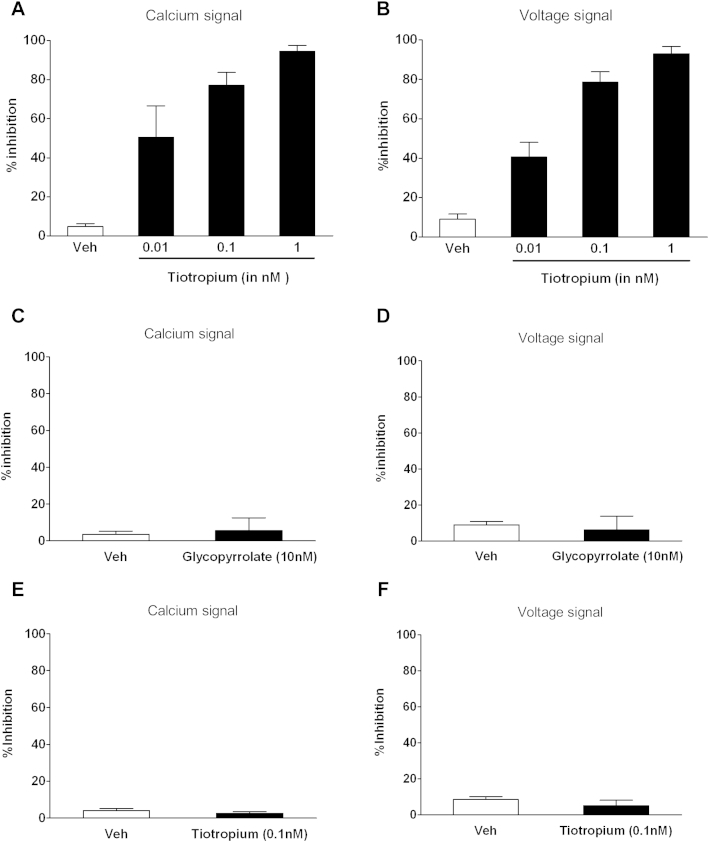
Effect of tiotropium and glycopyrrolate on capsaicin- and acrolein-stimulated primary airway guinea pig ganglion cells. **A** and **B,** Percentage inhibition caused by tiotropium of the calcium levels and voltage changes after capsaicin (0.1 μmol/L) challenge. **C** and **D,** Data from testing glycopyrrolate against capsaicin (0.1 μmol/L) challenge. **E** and **F,** Data from testing tiotropium against acrolein (10 μmol/L) responses. Data are shown as means ± SEMs of the percentage inhibition of tussive agent–induced depolarization (n = 4).

Although the vagus system has many benefits, one issue is that some of the sensory nerves contained in the trunk will innervate other nonlung organs, and thus the data obtained might not be indicative of airway-specific fibers. To address this, we harvested airway-specific primary ganglion neurons (stained with Dil; Fig 4, *A*) and assessed changes in [Ca^2+^]_i_ levels and membrane voltage. Tiotropium inhibited TRPV1-driven (Figs 4, *B*, and 5, *A* and *B*), but not TRPA1-driven, responses (Figs 4, *C*, and 5, *E* and *F*). Glycopyrrolate did not modulate capsaicin-induced increases in [Ca^2+^]_i_ levels or membrane voltage (Fig 5, *C* and *D*). Similar to the data generated in the isolated vagus, MCh did not trigger calcium movement or voltage changes in primary airway jugular neurons (see Fig E4 in this article's Online Repository at www.jacionline.org). Concentration responses to MCh on cells from the nodose ganglia were also performed to ensure that MCh does not activate other nerve fibers that innervate the airways. However, there was no effect on either the calcium signal or membrane depolarization at any concentration of MCh (data not shown). In general, a 10-fold lower concentration of test agents was required to reach the required response in these experiments compared with those on the vagal trunk.

### Effect of tiotropium on capsaicin-driven TRPV1 FLIPR assays (GenScript)

**Fig E5:**
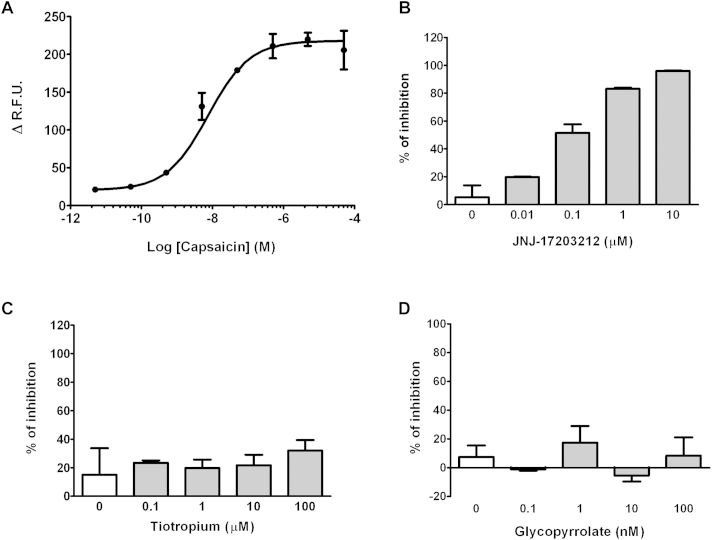
Effect of tiotropium on capsaicin responses in a FLIPR assay (work performed by using GenScript). HEK293 cells were genetically engineered to overexpress human TRPV1, plated at 20,000 cells per well, and stimulated with capsaicin. **A,** Concentration response curve with data expressed as relative fluorescence units *(R.F.U.)*. **B-D,** From these data, a submaximal concentration of capsaicin was chosen to profile the positive control, TRPV1 antagonist JNJ-172030212 (Fig 5, *B*), tiotropium (Fig 5, *C*), and glycopyrrolate (Fig 5, *D*). Experiments were run in duplicates.

To determine whether tiotropium was modulating TRPV1 function by acting as a classic receptor/ion channel antagonist, it was evaluated in a GenScript FLIPR assay. JNJ-17203212 (TRPV1 positive control), tiotropium, and glycopyrrolate were profiled against a submaximal concentration of capsaicin (10 nmol/L; selected from a response curve, see Fig E5, *A*, in this article's Online Repository at www.jacionline.org). JNJ-17203212 inhibited the capsaicin-induced signal in a concentration-dependent manner in HEK293 cells (see Fig E5, *B*); however, the other compounds did not significantly inhibit the signal under these assay conditions (see Fig E5, *C* and *D*).

### Effect of tiotropium on C-fiber activation by capsaicin *in vivo*

**Fig 6:**
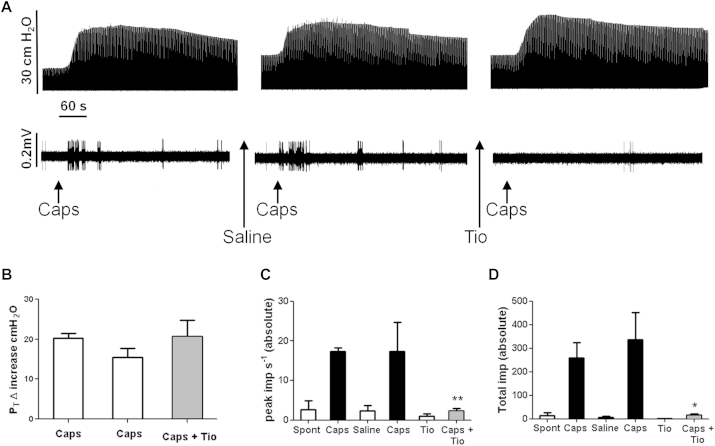
Effect of tiotropium on capsaicin-induced bronchospasm and firing of airway-specific sensory afferent nerves. **A,** Example of recording of bronchospasm *(top trace)* and simultaneous vagus nerve single C-fiber firing *(bottom trace)*. Capsaicin (100 μmol/L) was administered by means of aerosol for 15 seconds and indicated by *arrows* below the traces. Saline (0.9% for 1 minute) and tiotropium (100 μg/mL for 1 minute) administration are indicated below the traces by *arrows*. Voltage and pressure scales are indicated by *bars on the left of the traces*, and the time scale is provided by a 60-second *black bar* between the traces. **B-D,** Histograms represent the average changes in airway pressure induced by capsaicin before and after tiotropium (Fig 6, *B*), the average peak frequency of impulses per second recorded from vagus nerve single C-fiber capsaicin-induced firing before and after tiotropium (Fig 6, *C*), and the average total impulse count over time (5-minute window from nebulization) recorded from vagus nerve single C-fiber capsaicin-induced firing before and after tiotropium (Fig 6, *D*). Data are shown as means ± SEMs (n = 3). *P < .05 and **P < .01 as determined by Mann-Whitney *U* test.

Inhaled capsaicin (100 μmol/L) administered by means of aerosol for 15 seconds induced a burst of C-fiber firing and bronchospasm in anaesthetized guinea pigs (Fig 6). Fig 6, *A*, shows an example trace; the top panel illustrates changes in airway pressure (bronchospasm), and the bottom panel is the simultaneous measurement of airway nerve single C-fiber firing. Fig 6, *B*, depicts the average change in airway pressure after the various treatments; Fig 6, *C*, depicts the average peak frequency of impulses per second; and Fig 6, *D*, depicts the total impulses counted over the time recorded from a single C-fiber afferent treated with capsaicin. Pretreatment with tiotropium (100 μg/mL ≈ 212 μmol/L) reduced capsaicin-induced firing to a level not significantly different from spontaneous firing (an example is shown in the bottom trace of the far right part of Fig 6, *A*; and averages are shown in Fig 6, *C* and *D*). Interestingly, the increase in airway pressure did not appear to be altered (Fig 6, *B*).

### Effect of tiotropium on capsaicin-induced contractions of isolated guinea pig trachea

**Fig E6:**
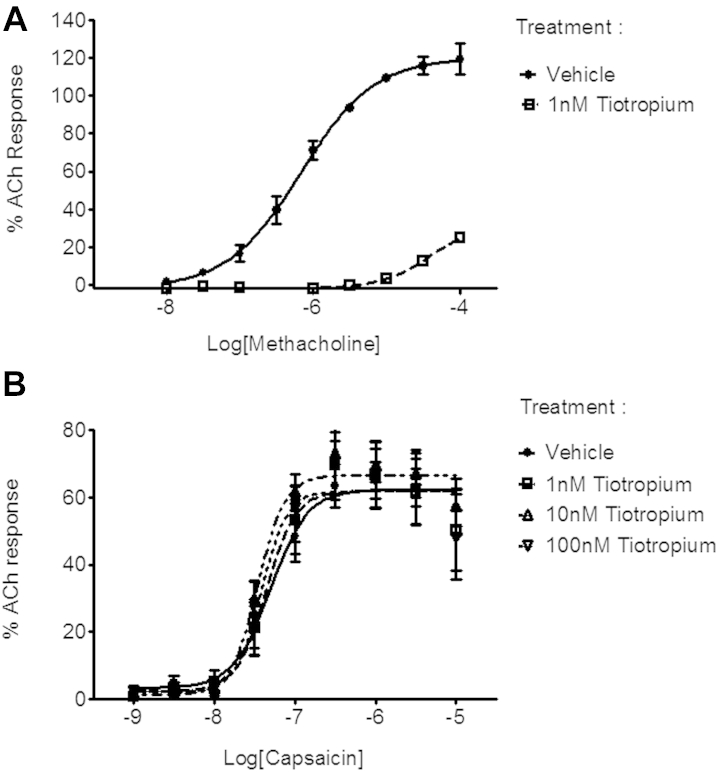
Effect of tiotropium on capsaicin-induced contraction of guinea pig trachea. The tracheal tissue was inserted into 10-mL tissue baths with gassed Krebs-Henseleit solution kept at 37°C. The tissue was placed under 1 g of tension and left to settle for 1 hour, with the Krebs-Henseleit solution being replaced occasionally. The tissue was exposed to a supramaximal concentration of acetylcholine (1 mmol/L) and then washed; this was repeated twice more. The tissues were then incubated with vehicle or tiotropium for 30 minutes, after which cumulative response curves were measured to MCh **(A)** or capsaicin **(B)**, and levels of tension in the tissue were recorded and expressed as a percentage of the average acetylcholine *(ACh)* response (n = 4).

MCh and capsaicin caused contraction of the isolated guinea pig trachea (see Fig E6 in this article's Online Repository at www.jacionline.org). Tiotropium caused a concentration-related inhibition of MCh responses (see Fig E6, *A*) but did not affect capsaicin-induced contractions (see Fig E6, *B*). The positive control JNJ-17203212 (100 μmol/L) blocked capsaicin responses (data not shown).

## Discussion

Bronchodilators provide the main pharmacologic intervention used in the treatment of COPD and are often used in the treatment of asthma. Anticholinergic agents appear to be the most effective therapy used to combat increases in airway bronchomotor tone.^2^ There are currently 2 anticholinergic agents available for the treatment of COPD: the short-acting drug ipratropium bromide and the LAMA tiotropium. However, some of the clinical benefit associated with taking these compounds (eg, the antitussive activity^10^) cannot easily be explained by their proposed mechanism of action as muscarinic receptor antagonists. In addition to bronchodilation, tiotropium and ipratropium have been shown to inhibit capsaicin-induced cough in animal models^21^ and in patients with URIs^10,22^ and asthma.^23^ Ipratropium has also been found to inhibit cough induced by ultrasonically nebulized distilled water in both healthy and asthmatic subjects.^24^ Furthermore, a systematic review of the literature assessing all trials in adult patients with respiratory and nonrespiratory diseases (excluding those with cancer who had chronic cough as a secondary or primary outcome) suggested that ipratropium possesses antitussive properties.^25^ These studies aimed to investigate the mechanism of action behind the inhibitory activity on sensory nerve activity and cough.

In these studies we have presented conclusive data that suggest, for the first time, that tiotropium inhibits TRPV1-mediated effects through a mechanism unrelated to its anticholinergic activity. Tiotropium significantly attenuated capsaicin-evoked cough at similar doses to that required to block MCh-driven bronchospasm, suggesting that this additional activity of tiotropium occurs at clinically relevant doses. Further evidence pointing to an interaction with the TRPV1 ion channel comes from data demonstrating an inhibitory activity on capsaicin-induced depolarization of guinea pig, rat, and human vagus nerve tissues. The lack of effect in mouse tissue indicates a likely species difference in the interaction of tiotropium with the TRPV1 ion channel expressed on the vagus nerve. Complementary data demonstrating an inhibitory activity of tiotropium on capsaicin-induced calcium influx and membrane voltage in primary jugular ganglion cells and action potentials in single airway C-fibers, as assessed *in vivo*, indicates an interaction with TRPV1 ion channels on airway-specific C-fibers.

Although the *in vivo* data obtained in the conscious cough model strongly suggest that tiotropium can modulate TRPV1 airway responses, it could be argued that the inhibition seen *in vivo* could occur through an effect on vagally mediated, muscarinic agonist–driven changes in airway tone, mucus production, or both. To avoid issues with data interpretation associated with *in vivo* studies, we used the isolated vagus nerve preparation to probe mechanistic questions in greater detail. This preparation allowed us to show that tiotropium inhibits sensory nerve depolarization induced by other stimuli known to activate sensory nerves totally or partially through TRPV1 (ie, RTX^15^ and the endogenous mediator PGE_2_).^13^ This general inhibitory activity on TRPV1-mediated activation of vagal afferents, irrespective of the ligand used, would suggest that these effects are not specific to capsaicin and further support an interaction with TRPV1. The similar inhibitory effect of the structurally similar ipratropium^26^ and the lack of an inhibitory effect with atropine or a second LAMA, glycopyrrolate,^27-29^ suggest that modulation of TRPV1 is particular to certain antagonists rather than through their common mechanism of action of muscarinic receptor antagonism. The lack of inhibitory activity of atropine and glycopyrrolate on capsaicin–induced vagal depolarization is in contrast to data suggesting that these compounds can inhibit capsaicin-induced cough in animal models^30-32^ and in a limited data set showing this activity in a clinical healthy volunteer study.^33^ This might suggest that it is indeed the anticholinergic activity of these drugs that confers this antitussive activity or our favored explanation in light of these data that any inhibitory action observed on tussive responses is mediated through alternative mechanisms.

Further evidence to suggest that the activity of tiotropium on sensory afferents is not through its activity at muscarinic receptors comes from the fact that an exogenous muscarinic agonist failed to cause depolarization of the vagal tissue. We were also able to demonstrate that the activity of tiotropium appears to be specific for TRPV1-driven responses because it does not modulate the depolarization evoked by TRPA1 or TRPV4 agonists. Finally, we were able to test the hypothesis in vagal tissue harvested from human donor tissue surplus to transplant requirement. Depolarization of the isolated human vagus has previously been shown to be similar to that seen in guinea pigs and predictive of cough.^11-14^ The inhibitory activity of tiotropium on capsaicin-induced depolarization in this assay provides translational data that support our hypothesis.

In parallel, many of our key findings in isolated vagus nerves were duplicated in isolated primary jugular airway ganglion neurons. This system has the added benefit of being able to identify and study airway-specific nerve cells. This is important given the vagal trunk contains nerves connected to an array of internal organs. The data obtained showed that tiotropium, but not another LAMA, glycopyrrolate, was a potent modulator of TRPV1 (capsaicin)–induced but not TRPA1 (acrolein)–induced changes in calcium and membrane voltage. Furthermore, MCh did not change calcium levels or membrane voltage in airway sensory neurons, which is consistent with the theory that tiotropium is not behaving as a classical muscarinic receptor antagonist in this context.

Interestingly, tiotropium did not significantly inhibit increases in calcium levels evoked by capsaicin in a commercial FLIPR assay (approximately 20% reduction in the peak signal, GenScript). It was not immediately clear why there was a discrepancy between data generated in our primary cell and tissue assays and that generated in a configured system using a cell line that is genetically altered to overexpress human TRPV1 channels. However, the positive control (JNJ-17203212), a well-known TRPV1 antagonist,^34^ performed as one would expect. Indeed, the data obtained were very similar to those we have published previously using the isolated vagal system, both in respect to potency and efficacy.^13^ Thus we suggest that tiotropium does not act as a “typical” TRPV1 antagonist and acts either by interacting with another binding site on the channel or by acting indirectly as a modulator of the TRPV1 channel. Further evidence for this “nontypical” interaction is the apparent lack of effect of tiotropium on capsaicin-induced contraction of isolated guinea pig trachea and bronchospasm induced in the *in vivo* single-fiber recording model in which tiotropium reduced capsaicin-induced afferent fiber firing without modulating the changes in bronchospasm. The contraction/bronchospasm observed in these 2 systems is thought to be through capsaicin acting on TRPV1 channels on nerves and causing the local release of substance P, which then acts on neurokinin receptors on the airway smooth muscle causing it to contract.^35,36^ A classical TRPV1 antagonist would be expected to modulate these responses; indeed, here we have shown that JNJ-17203212 inhibits capsaicin-induced contraction in guinea pig airways. Identifying this off-target but beneficial action of tiotropium on sensory nerve activation could open up other therapeutic areas for this compound, including the treatment of cough and the sensory hyperresponsiveness phenotype, which might be involved in dyspnea, the late asthmatic response, and airway hyperresponsiveness.^37-39^

Interestingly, the use of tiotropium in patients has not been associated with the unwanted side effects associated with TRPV1 antagonists (ie, hyperthermia).^40^ The fact that it is not a classical antagonist could explain why no one has observed these effects. Other explanations could be that the route of administration (ie, topical) and dosing regimen (ie, once a day) could reduce the thermal effects. Another possibility could be that tiotropium has a selectivity profile similar to that of many of the TRPV1 antagonists recently described that are not reported to have effects on temperature control^41-44^ or because the change in thermal control has been very transient, as has recently been observed in a phase 1 clinical trial with a new TRPV1 antagonist.^45^

In conclusion, for the first time, we have shown that tiotropium inhibits TRPV1-mediated effects through a mechanism unrelated to its anticholinergic activity. The activity of tiotropium on TRPV1 might be responsible, at least in part, for some of the clinical benefit associated with taking it and might suggest alternative applications for this compound outside of COPD in respiratory and nonrespiratory disorders in which cough is a major debilitating symptom. Several other LAMAs (eg, aclidinium bromide^46^) are currently in clinical development, and it remains to be seen whether these compounds share the same beneficial properties.

**Key messages**

• Recent studies have suggested that the LAMA tiotropium, a drug widely prescribed for its bronchodilator activity in patients with COPD and asthma, improves symptoms and attenuates capsaicin-induced cough in preclinical and clinical challenge studies.
• For the first time, we have demonstrated that tiotropium inhibits TRPV1-mediated effects in sensory afferents through a mechanism unrelated to its anticholinergic activity.
• We suggest that some of the clinical benefit associated with taking tiotropium could be explained through this proposed mechanism of action.

## Methods

### Effect of tiotropium on capsaicin-induced cough

To establish an effective dosing regimen, we first performed a concentration response to inhaled tiotropium against MCh (muscarinic receptor agonist)–induced bronchospasm. Conscious male Dunkin-Hartley guinea pigs (350-450 g; Harlan, Bicester, United Kingdom) were placed in an acrylic glass chamber and exposed to either aerosolized vehicle (0.5% ethanol in saline) or tiotropium (3, 10, or 30 μg/mL; this equates to 6.35, 21.2, and 63.5 μmol/L solution; Boehringer Ingelheim, Ingelheim, Germany) for 10 minutes. Fifty minutes later, the guinea pigs were placed in the same chambers used to record cough and challenged with either vehicle (saline) or a submaximal dose of inhaled MCh (0.1 μg/mL). We estimate that over the 10 minutes of challenge, the lungs will be exposed to approximately 0.28 to 2.8 μg of tiotropium (based on a breathing rate of 80 breaths/min, a tidal volume of 3.5 mL, and a deposition rate of 10% with the aerosolized 10 μg/mL solution, which is “diluted” 1,000- to 10,000-fold with room air from the nebulizer system). Changes in Penh (an estimate of bronchospasm) were recorded for 5 minutes. From these data, doses of tiotropium were selected to be tested against capsaicin-induced cough. Another set of guinea pigs was dosed (as above) with vehicle (saline) or tiotropium (10 or 100 μg/mL) 60 minutes before exposure to aerosolized capsaicin (60 μmol/L) for 5 minutes. The number of coughs was recorded during aerosol challenge and for 5 minutes after challenge and confirmed by a trained observer blinded to prior treatment, as previously described.^E1-E3^ Experiments were approved by the Imperial College London ethical review process committee, and procedures strictly adhered to the Animals (Scientific Procedures) Act 1986 UK Home Office guidelines. Experiments were performed under a Home office project license (PPL 70/7212).

### Effect of tiotropium on isolated vagal sensory nerve tissue

This preparation has previously been characterized and shown to respond to a range of agents known to cause cough both in preclinical models and in human cough studies and provides the opportunity to conduct a comprehensive pharmacologic assessment of the direct action of drugs on the vagus nerve without the pharmacokinetic and numerous other considerations that limit the interpretation of *in vivo* data.^E1-E3^ In addition, the isolated sensory nerve system allows us to perform parallel experiments with human tissue, thus generating translational data.^E4^ Vagal tissue was harvested from male Dunkin-Hartley guinea pigs, male Brown Norway rats, or male C57/Bl6 mice or through donor human lung tissue acquired through the International Institute for the Advancement of Medicine (Edison, NJ; consent given for scientific research), and experiments were conducted, as previously described. Human tissue was obtained from 5 donors (4 male subjects age 36-57 years and 1 female subject age 45 years) who had no known respiratory disease.

The effect of tiotropium was investigated on depolarization induced by a range of TRPV1 agonists, including capsaicin (1 μmol/L), RTX (3 nmol/L), and PGE_2_ (10 μmol/L), which is an endogenous mediator known to work partially through TRPV1.^E3,E5^ Other muscarinic receptor antagonists were also profiled against capsaicin-induced depolarization, including ipratropium, atropine, and the LAMA glycopyrrolate.^E6-E8^

Tiotropium was tested against depolarization induced by the TRPA1 agonist acrolein (300 μmol/L)^E2^ and the TRPV4 agonist GSK1016790A (0.3 μmol/L) to determine whether the effect of tiotropium was specific to TRPV1-induced responses or whether it had a general effect on sensory nerves.^E9^ Key experiments were repeated with human vagal tissue. To further investigate whether the effect of tiotropium was through its anticholinergic activity, we determined whether exogenous MCh would depolarize the vagal preparation. We selected appropriate concentrations of MCh using an isolated guinea pig tracheal system to assess contraction, as previously described.^E10^

### Effect of tiotropium on airway-specific ganglion cells

Identification of airway-specific neurons was performed, as previously described.^E11,E12^ Briefly, 14 days before the experiment, guinea pigs were dosed intranasally with the lipophilic retrograde tracer dye DiI (1 mL/kg of 625 μg/mL; see compounds and materials). Guinea pigs were then killed, and the jugular ganglia were harvested to measure calcium movement and membrane voltage change, as described previously.^E3^ Ganglia were predigested with collagenase/Dispase II at 37°C. Ganglia were then digested with papain for 30 minutes with gentle agitation at 37°C and collagenase IV/Dispase II for 40 minutes at 37°C. The cells were then separated and placed onto laminin-coated plates and flooded with complete F12 media after adherence. DiI-labeled neurons from the jugular ganglia were then stained with both a ratiometric calcium-sensitive dye (Fura2-AM, 3 μmol/L) and a voltage-sensitive dye (Di-8-ANEPPS). Responsiveness and viability of neurons were assessed by means of application of K_50_ at the start and end of recording. [Ca^2+^]_i_ responses were recorded as the area under the curve, and membrane depolarization responses were recorded as the peak magnitude. All responses were normalized to the initial K_50_ application so that one could account for differences between individual cell responses. Tiotropium and glycopyrrolate were evaluated against capsaicin (0.1 μmol/L)–induced and acrolein (10 μmol/L)–induced responses. In addition, we evaluated the effect of MCh on [Ca^2+^]_i_ levels and voltage changes.

### Effect of tiotropium on capsaicin-driven TRPV1 FLIPR assays (performed with GenScript)

HEK293 cells genetically modified to overexpress human TRPV1 were seeded in a 384-well, black-wall, clear-bottom plate at a density of 20,000 cells per well in 20 μL of medium. Cells were cultured for 18 hours before the day of the experiment and maintained at 37°C and 5% CO_2_. Capsaicin concentration-response curves were performed to select a submaximal concentration. Calcium-4 in conjunction with a Fluorescent Imaging Plate Reader was used to record the signal. Vehicle, tiotropium, glycopyrrolate, or the positive control JNJ-17203212 (TRPV1 antagonist^E3,E13^) were added to the well and incubated at 37°C for 60 minutes before the addition of capsaicin (10 nmol/L). Assays were performed in duplicate.

### Effect of tiotropium on capsaicin-induced firing of single-fiber afferents and bronchospasm

Guinea pigs were anesthetized with urethane (1.5 g/kg) intraperitoneally. If required, anesthesia was supplemented with additional urethane. The trachea was cannulated, and bronchospasm was measured with an air-pressure transducer (SenSym 647, Farnell, United Kingdom) connected to a side arm of the tracheal cannula. Blood gases and pH were maintained at physiologic levels by means of artificial ventilation (Ugo Basile small-animal ventilator, Varese, Italy), with a tidal volume of 10 mL/kg and 50 to 60 breaths per minute of laboratory air. The right jugular vein and carotid artery were cannulated for injecting drugs and measuring systemic arterial blood pressure (Gould P23XL transducer, Redruth, United Kingdom), respectively. Body temperature was continuously monitored with a rectal thermometer and maintained at 37°C with a heated blanket and control unit (Harvard Apparatus, Holliston, Mass). Animals were paralyzed with vecuronium bromide that was initially administered at a dose of 0.10 mg/kg intravenously and followed every 20 minutes with 0.05 mg/kg administered intravenously to maintain paralysis. The depth of anesthesia was frequently assessed by monitoring the response of heart rate and blood pressure to noxious stimuli. Both cervical vagus nerves were located through a cervical incision and dissected free from the carotid artery and sympathetic and aortic nerves; both vagus nerves were cut at the central end, posterior to the vagal ganglia. The left vagus nerve was used for sensory nerve fiber recording and was cleared of its surrounding fascia. The skin and muscle in the neck at either side of the incision were lifted and tied to a metal ring to form a well, which was filled with light mineral oil. Bipolar Teflon-coated platinum electrodes (exposed at the tips) were used for recording purposes by using fascia positioned on one electrode for a reference. The vagus nerve was placed on a small black acrylic glass plate to facilitate subsequent dissection. Thin filaments of nerve were teased from the vagus nerve under a binocular microscope and placed on the second electrode until a single active unit or 1 of not more than 2 or 3 units was obtained. Action potentials were recorded in a conventional manner by using electrodes connected to a pre-amp headstage (Digitimer NL100K, Welwyn Garden City, United Kingdom). The signal was amplified (x5000, Digitimer NL104), filtered (LF30Hz-HF8.5kHz, Digitimer NL125), and passed through a Humbug noise reducer (AutoMate Scientific, Berkeley, Calif) before input sampling and recording. All signals were sampled (50 kHz) and recorded with the Spike 2 software data acquisition system through a CED Micro1401 interface (Cambridge Electronics Design, Cambridge, United Kingdom). The software allowed pulse train counting over selected time periods. In addition, monitoring of the input signal to the Spike software was also carried out on a digital storage oscilloscope (Tektronix DPO 2012, Bracknell, United Kingdom). The input signal was also fed through an audio amplifier to a loudspeaker. All animals were killed at the end of the experiments with an overdose of pentobarbitone.

Conduction velocities were measured to distinguish slow-conducting nonmyelinated C-fibers from fast-conducting myelinated A-fibers by stimulating the vagus nerve close to the thorax with bipolar silver electrodes using a suprathreshold voltage at 0.5 ms and 1 Hz (Grass stimulator, Warwick, RI). The corresponding action potential was recorded in the nerve fiber under observation. The stimulus and the recorded action potential were captured on the Spike software in a single sweep, and the time interval between them was measured to calculate the velocity by using the distance from the cathode-stimulating electrode and the recording electrode. Aerosols were generated by an Aerogen nebulizer (Buxco Nebulizer Control, Wantage, United Kingdom) connected to the ventilator and arranged so that the inspired air passed through the medication chamber before entering the lungs of anesthetized animals through the tracheal cannula. Single vagus nerve fibers were identified as originating from the 3 groups of airway sensory nerve endings (ie, slowly adapting stretch receptors, irritant receptors [rapidly adapting stretch receptors, RARs, Aδ-fibers], and pulmonary/bronchial C-fiber receptors) by using several criteria.^E14^ These included pattern of spontaneous discharge, response to hyperinflation and deflation, adaptation indices, and response to capsaicin aerosol administration and conduction velocities. As a rule, a fiber that had no obvious pattern to the spontaneous activity (often very sparse), did not respond to hyperinflation or hyperdeflation, and responded to capsaicin aerosol was considered for further investigation. Finally, verification of a C-fiber was confirmed at the end of the experiment by determining conduction velocity. After surgery, the animals were allowed to stabilize for at least 30 minutes. After identification of a lung afferent fiber and its sensitivity to capsaicin aerosol, the following protocol was pursued: after a control baseline recording of at least 2 minutes, capsaicin (100 μmol/L) was administered by means of aerosol for 15 seconds, and the changes in fiber activity, intratracheal pressure, and blood pressure were continuously recorded until baseline or a steady state was re-established. After an interval of 10 minutes, saline (aerosol for 60 seconds) was administered while recording variables. After another interval of 10 minutes, the capsaicin aerosol was repeated. Ten minutes later, tiotropium (100 μg/mL for 60 seconds) was administered by means of aerosol, followed 30 minutes later by the capsaicin aerosol.

### Effect of tiotropium on isolated guinea pig tracheal contractions

Contractile responses to MCh or capsaicin were induced in isolated guinea pig trachea by using a system previously described by Buckley et al.^E10^ The effect of tiotropium was determined against MCh and capsaicin; the TRPV1 antagonist JNJ-17203212 (100 μmol/L)^E13^ was included as a positive control in the capsaicin experiments.

Pharmacologic agents, such as the anesthetics used in the animal experiments (eg, pentobarbitone, urethane, and vecuronium bromide) and any of the drugs transplant donors had been taking before organ harvesting, could be affecting sensory nerve responses in these systems. However, we are confident that they would not complicate interpretation of the data with tiotropium because we have time-matched, vehicle-treated control responses that will also have been influenced by such agents. Furthermore, we believe that as the ganglion cells are harvested through a complex multistep extraction process and the isolated vagal preparation is constantly perfused, any anesthetic agents, for example, will be removed before the start of assessment.

### Compounds and materials

#### *In vivo* cough experiments

Capsaicin (Sigma, Dorset, United Kingdom) was prepared as a 10 mmol/L stock made in ethanol and was diluted in vehicle to obtain a concentration of 60 μmol/L with 1% ethanol and 1% Tween 80 in 0.9% saline. Tiotropium (Boehringer Ingelheim) was prepared at concentrations of 10, 30, or 100 μg/mL with 0.5% ethanol in 0.9% saline. MCh was prepared in 0.9% saline.

#### *In vivo* single-fiber experiments

Capsaicin (Sigma-Aldrich) was dissolved in 1% ethanol and 1% Tween 80 in 0.9% saline to working solution. Tiotropium (Boehringer Ingelheim) was dissolved in 0.9% saline. Vecuronium bromide (Sigma-Aldrich) was dissolved in distilled water at 2.5 mg ∙ mL^−1^ stock and diluted in distilled H_2_O, as required.

#### *In vivo* staining of airways neurons

The lipophilic retrograde tracer dye DiI (Invitrogen, Carlsbad, Calif) was prepared as a stock of 25 mg/mL diluted in 100% ethanol and kept in the dark at room temperature. On the day of staining, the dye was diluted in 0.9% sterile saline at a concentration of 625 μg/mL. A dose of 1 mL/kg was used for staining.

#### *In vitro* experiments

Capsaicin, RTX, MCh, glycopyrrolate, atropine, acrolein, GSK1016790a, and ipratropium were purchased from Sigma-Aldrich. PGE_2_ was purchased from Cayman Chemical (Ann Arbor, Mich). HC-030031 was purchased from Chembridge (San Diego, Calif), and HC-067047 was purchased from Peakdale Molecular Ltd (High Peak, United Kingdom). Tiotropium was a gift from Boehringer Ingelheim (Germany), and JNJ-17203212 was a gift from GlaxoSmithKline. All compound stock solutions were prepared in 100% dimethyl sulfoxide (DMSO), except for tiotropium, ipratropium, and MCh, which were prepared on the day of experiment in Krebs-Henseleit solution (for vagus nerve experiments) or distilled water (for imaging), and PGE_2_, which was dissolved in ethanol. On the day of the experiment, the compounds were diluted 1:1000 in the appropriate medium to obtain the desired final concentrations.

#### *In vitro* vagus nerve experiments

Krebs-Henseleit solution (NaCl, 118 mmol/L; KCl, 5.9 mmol/L; MgSO_4_, 1.2 mmol/L; CaCl_2_, 2.5 mmol/L; NaH_2_PO_4_, 1.2 mmol/L; NaHCO_3_, 25.5 mmol/L; and glucose, 5.6 mmol/L) was made fresh on a daily basis. All salts were purchased from BDH (Dorset, United Kingdom). On the day of the experiment, stocks were diluted in Krebs-Henseleit solution to obtain the desired concentration with 0.1% DMSO.

#### *In vitro* imaging experiments

Extracellular solution (ECS; 5.4 mmol/L KCl, 136 mmol/L NaCl, 1 mmol/L MgCl_2_, 2.5 mmol/L CaCl_2_, 0.33 mmol/L NaH_2_PO_4_, 10 mmol/L D-glucose, and 10 mmol/L HEPES; 297 mOsm; pH adjusted to 7.4 with NaOH at 37°C) and K_50_ (50 mmol/L KCl, 101.4 mmol/L NaCl, 1 mmol/L MgCl_2_, 2.5 mmol/L CaCl_2_, 0.33 mmol/L NaH_2_PO_4_, 10 mmol/L D-glucose, 10 mmol/L HEPES; 297 mOsm; pH adjusted to 7.4 with NaOH at 37°C) solutions were made fresh daily. All salts were from BDH and Sigma-Aldrich. Di-8-ANEPPS and Fura2-AM stocks were prepared as 10-μL aliquots in DMSO and frozen at −20°C. To load the cells, Di-8-ANEPPS and Fura2-AM aliquots were diluted in 2.5 and 1 mL ECS, respectively. Compound stock solutions were diluted 1:1000 in ECS to the desired final concentrations with 0.1% vehicle (DMSO or distilled water).

### Data analysis and statistics

Inhibition of capsaicin-induced cough was analyzed by using the nonparametric Kruskal-Wallis test with the Dunn *post hoc* test, comparing drug group responses with those of the vehicle control group. Data are presented as medians ± interquartile ranges. In the *in vivo* single-fiber experiments, inhibition was determined by using a 2-tailed paired Student *t* test, comparing responses to agonist (in the same fiber) in the presence and absence of vehicle/antagonist. Inhibition of agonist-induced vagus nerve depolarization was analyzed by using the 2-tailed paired Student *t* test, comparing responses to agonist (in the same piece of vagus nerve) in the absence and presence of vehicle/antagonist. For imaging, all responses in each cell were normalized to the initial response generated by application of K_50_ within the same cell. Data were analyzed for “responding” cells only, which were defined as neurons with a response of 10% or greater of K_50_. Inhibition of responses was analyzed by using the 2-tailed Student paired *t* test, comparing responses to agonist (in the same cell) in the absence and presence of vehicle/antagonist. Data are presented as means ± SEMs. For all statistical analyses, *P* values of less than .05 were considered significant.
